# Impact of pre-procedural requirements on time to aortic valve replacement: Transcatheter AVR vs surgical AVR^☆^

**DOI:** 10.1016/j.ahjo.2025.100698

**Published:** 2025-12-10

**Authors:** Curtiss Stinis, Sean Tunis, Sandra Lauck, Aakriti Gupta, Shannon Murphy, Soumya Chikermane, Seth Clancy, Mark Russo

**Affiliations:** a Scripps Clinic John R. Anderson V Medical Pavilion, La Jolla, CA, USA; b Rubix Health, Baltimore, MD, USA; c Centre for Heart Valve Innovation, St. Paul's Hospital, University of British Columbia, Vancouver, British Columbia, Canada; d Cedars-Sinai Medical Center, Smidt Heart Institute, Los Angeles, CA, USA; e Edwards Lifesciences, USA; f Division of Cardiac Surgery, Rutgers-Robert Wood Johnson Medical School, New Brunswick, NJ, USA

**Keywords:** Aortic stenosis treatment, TAVR procedure, SAVR procedure, Cardiac surgery, Heart surgery delays, Cardiac surgery prerequisites

## Abstract

**Background/objective:**

This study aims to understand the extent that cardiac specialist visits and imaging requirements contribute to the difference in time to Aortic valve replacement (AVR) stratified by approach transcatheter AVR (TAVR) and surgical AVR (SAVR).

**Methods:**

Optum Market Clarity Data was used to identify patients with clinically significant AS (CSAS) who received an AVR between 2016 and 2023 and whose AVR occurred within two years of their CSAS diagnosis. Patient characteristics were measured at baseline; pre-procedural factors, including the number of cardiac specialist visits and imaging events, were measured from CSAS diagnosis to AVR (TAVR vs SAVR). Stepwise generalized linear models were used to assess whether the number of cardiac specialist visits and imaging events contribute to the differences in time to TAVR and SAVR, after adjusting for baseline characteristics.

**Results:**

Of the 14,225 patients in the cohort, 42 % received a TAVR. Compared to the SAVR cohort, the TAVR cohort was, on average, more male, older, sicker, and had more Medicare enrollees. TAVR patients had approximately two times more cardiac specialist visits (3.73 vs 6.37) and imaging events (1.18 vs 2.07) than SAVR patients. Time to TAVR is 65 days longer (RR = 1.77, 1.67–1.87) than SAVR, after adjustment for patient characteristics. This difference reduces to 11 days (RR = 1.12, 1.07–1.17) after accounting cardiac specialist encounters and imaging events.

**Discussion:**

Pre-procedural encounters significantly contribute to the longer time to AVR for TAVR patients. Findings suggest a need for streamlining the pre-procedural process for TAVR to enhance timely care delivery for CSAS patients.

## Highlights

• TAVR patients waited approximately 59 days longer than SAVR (157 vs 98), risking outcomes for older, high-risk patients.
• TAVR patients underwent nearly twice as many cardiac specialist visits and imaging procedures compared to SAVR patients. These additional pre-procedural requirements account for 83% of the delay difference between treatment approaches.
• The significantly longer time to TAVR versus SAVR was consistent across age, sex, race, region, and payer groups.
• Simplifying requirements for routine TAVR procedures could reduce treatment delays, potentially improving patient outcomes.

## Graphical abstract

**Figure:**
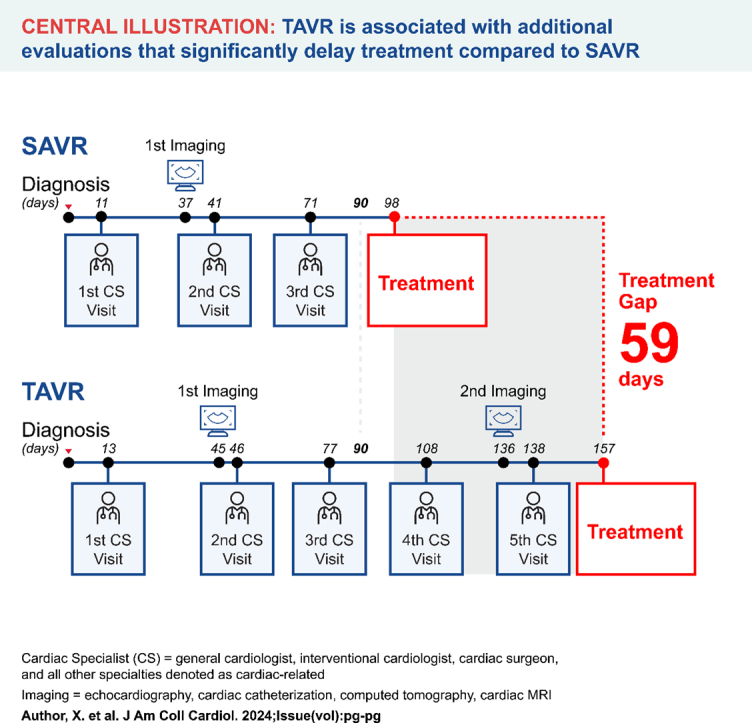

## 1 Introduction

Timely intervention in aortic stenosis (AS) is paramount, as delays in aortic valve replacement (AVR) can lead to significantly worsened outcomes, including higher mortality rates, irreversible myocardial damage [1,2], and a greater risk of sudden cardiac death [3,4]. Studies indicate that the risk of sudden cardiac death in asymptomatic patients with severe AS who are managed conservatively is approximately 1 % per year [[5], [6], [7]]; this risk can increase to 12 % within six months after the onset of symptoms if intervention is delayed [8]. Once symptoms develop, the clinical deterioration can be rapid, with substantial risks of sudden death occurring while awaiting intervention. Additionally, delaying AVR until patients exhibit advanced left ventricular decompensation not only increases perioperative mortality to as high as 19 % but also leaves patients with a poor long-term prognosis due to irreversible myocardial scarring that can develop while awaiting treatment [9].

Despite the critical need for timely AVR, delays in treatment remain common. Multiple studies have documented that these delays are associated with increased mortality, morbidity, and healthcare costs. For instance, Roule et al. demonstrated that longer wait times independently increased one-year mortality after successful TAVR by 2 % per week [10]. Similarly, Albassam et al. found that patients with severe AS awaiting intervention faced increasing wait times for both SAVR and TAVR, with associated increases in mortality and hospitalizations related to heart failure [11].

While the consequences of delayed AVR are well-established, there remains a critical gap in our understanding of the specific factors that contribute to these delays, particularly the disparity in wait times between TAVR and SAVR patients. Transcatheter aortic valve replacement (TAVR) has transformed the management of AS, especially in high-risk patients, but its comprehensive pre-procedural process may contribute to wait longer times compared to surgical aortic valve replacement (SAVR). However, the extent to which these pre-procedural requirements—such as additional imaging studies and specialist evaluations—directly contribute to treatment delays remains poorly quantified.

This study seeks to bridge this knowledge gap by investigating the specific pre-procedural factors that contribute to the difference in time to treatment between TAVR and SAVR. By identifying and quantifying these factors, we aim to provide actionable insights that could inform targeted interventions to streamline the pre-procedural process for AVR, ultimately enhancing timely care delivery and improving outcomes for patients with severe AS.

Given the critical importance of timely intervention for AS and the need to understand factors contributing to delays, we undertook a comprehensive analysis of real-world data to examine this issue in detail.

## 2 Methods

### 2.1 Study population

The Optum® de-identified Market Clarity Dataset (claims + EMR) dated 2007–2023Q3 is a robust, clinically rich, and payer-complete longitudinal integrated dataset that deterministically links medical and pharmacy claims with electronic health records (EHR). Natural language processing was applied to unstructured clinical notes across 100 million patient histories. Variables included, but were are not limited to, in-office assessments and measurements, diagnosis codes, signs and symptoms, biomarkers, labs, and test results. Since this study used de-identified (claims + EMR) data, informed consent was not required under an IRB exemption status. All aspects of this study were conducted in compliance with the Health Insurance Portability and Accountability Act of 1996 (HIPAA) regulations and the HIPAA Omnibus Rule of 2013.

For our study population, adults (18+) with clinically significant AS admitted for AVR (isolated or with concomitant procedures) between 1/2016 and 9/2023 were included. “Clinically significant” AS was defined as symptomatic AS, meaning that the patient had to have either (1) a diagnosis of heart failure (HF), defined as a record of any one of the ICD10 diagnosis codes for HF (I50 codes and I09.81, I11.0, I13.0 and I13.2), or (2) a record of at least two symptoms that occurred within ±30 days of an AS diagnosis. For the two symptoms, patients could have two different symptoms on the same day or the same symptom documented on two different days; the symptoms could be either by claims (via diagnosis codes) or EHR (via diagnosis codes or physician notes). The clinical symptoms that met this inclusion criterion were classified as e.g.: (1) chest discomfort (e.g., angina pectoris, exertional angina, resting angina, chest pressure, or precordial chest pain), (2) dyspnea (e.g., shortness of breath, dyspnea, paroxysmal nocturnal dyspnea, labored breathing, breathlessness, paroxysmal dyspnea), (3) dyspnea on exertion (dyspnea on exertion or effort), (4) syncope/presyncope (e.g., syncope, syncope and collapse, exertional syncope, fainting, fainting spell, presyncope), or (5) fatigue (fatigue considered severe).

**Fig. 1:**
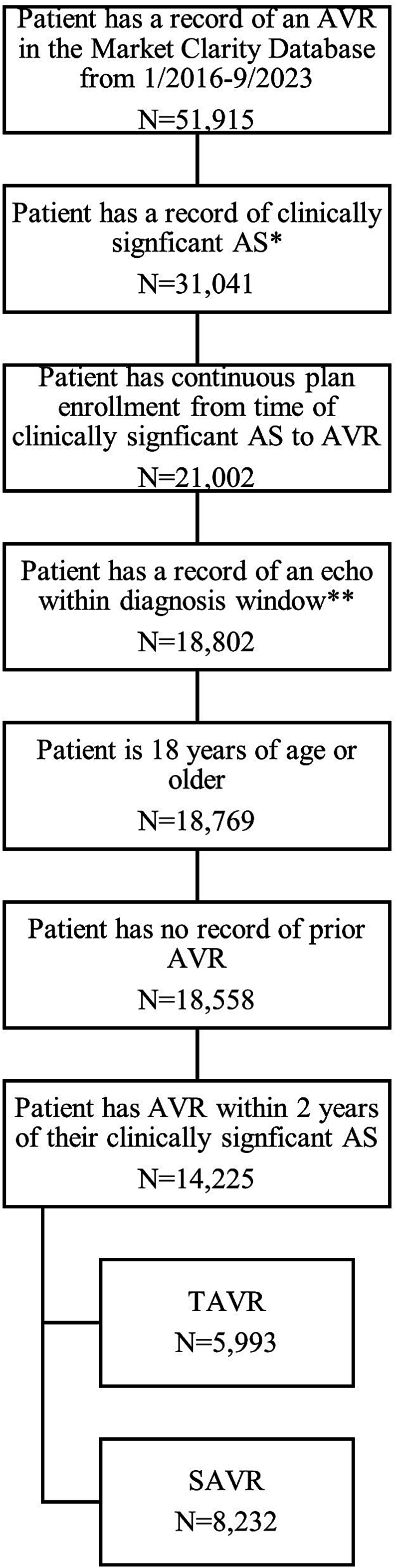
Cohort attrition diagram. *Clinically significant AS is defined as symptomatic AS, which means that the patient had either a diagnosis of heart failure OR a record of at least 2 of the following symptoms occuring within ±30 days of an AS diagnosis: chest pressure, dyspnea, dyspnea on exertion, syncope, fatigue. **Echo within diagnosis window defined as echo in the 30 days before the first AS diagnosis through the clinically significant AS date.

The clinically significant AS date was defined as the latter of the first AS diagnosis date and the first HF or symptomatic date. Our study required documented evidence of clinically significant AS with an identifiable diagnosis date to calculate time to AVR; this criterion was met by 60 % of patients with AVR records in the database. The population was limited to patients who received AVR within two years of the clinically significant AS date as a proxy for severity. Additionally, patients must have had continuous health plan enrollment (i.e., commercial, Medicare Advantage, Medicaid) within the six months prior to the clinically significant AS date through AVR, as well as a record of an echocardiogram within the 30 days prior to the first AS diagnosis through the clinically significant AS date. Patients with prior AVR were excluded. Fig. 1 provides an attrition diagram.

### 2.2 Cohorts and covariates

Data collected included AVR type (TAVR or SAVR), age, sex, race, region within the United States (Midwest, Northeast, South, West), payor (commercial, Medicare, other), year of clinically significant AS diagnosis, risk characteristics (Elixhauser Comorbidity Score and Hospital Frailty Risk Score [HFRS]), cardiac specialist referral (cardiology, cardiovascular surgery/surgeon, thoracic surgery/surgeon, thoracic/cardiovascular surgeon, cardiovascular disease specialist, interventional cardiology), cardiology visits, and imaging visits (echocardiography, cardiac catheterization, computed tomography, cardiac magnetic resonance imaging). Missing values for sex, race, region, and payor were imputed with the mode. For payor, the mode was determined by age category (commercial for age < 65 and Medicare for age 65+). For patients of Hispanic ethnicity, their race was reported as Hispanic, regardless of their documented race.

### 2.3 Statistical analyses

Descriptive statistics were generated for all variables of interest for the total population of both TAVR and SAVR patients, including unadjusted curves for time from clinically significant AS to TAVR and SAVR. General linear regression models (GLM) with gamma distribution and log link were used to account for the right-skewed distribution of time to AVR for the following four models: (1) covariates demographics and risk factors only, (2) covariates demographics and risk factors + cardiac specialist visits, (3) covariates demographics and risk factors + imaging, and (4) covariates demographics and risk factors + cardiac specialist visits + imaging. Stratified analyses of time to AVR by AVR type included: age, sex, race, region, payor, treated by cardiac specialists. Using these methodological approaches, we identified several key findings regarding the time to AVR and the factors influencing these timelines.

## 3 Results

### 3.1 Cohort attrition diagram

Fig. 1 provides the study attrition flowchart. In the Market Clarity database, there were 51,915 patients with an AVR record from 1/2016–9/2023. Approximately 60 % of the sample patients (31,041) met the requirement of having a record of clinically significant AS. After additional inclusion/exclusion criteria were applied (record of an echocardiogram, 18 years of age or older, continuous health plan enrollment six months before the clinically significant AS date through AVR, a record of an echocardiogram within the 30 days before the first AS diagnosis through the clinically significant AS date, and no record of prior AVR), the sample was reduced to 18,558 patients. Of these, 39 % of TAVR patients and 62 % of SAVR patients received their AVR within 90 days of their clinically signficant AS diagnosis, and at two years 68 % of TAVR patients and 84 % of SAVR patients had received their AVR. After excluding patients not treated within two years, the final sample for analyses was 14,225 AVRs (5993 TAVRs and 8232 SAVRs).

### 3.2 Time from clinically siginficant AS to SAVR and TAVR

**Fig. 2:**
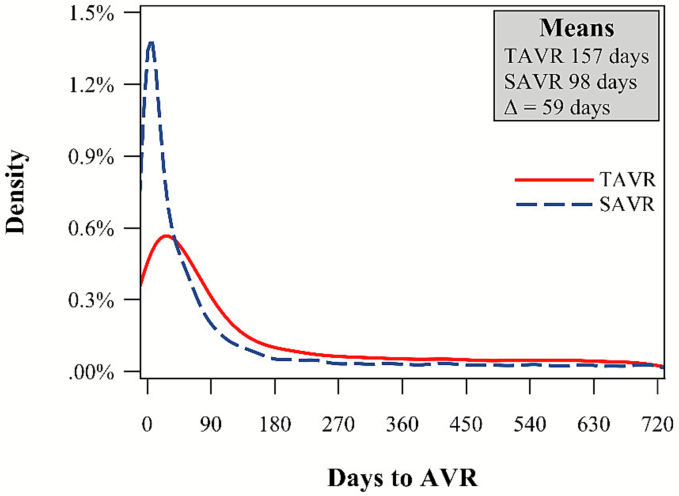
Time from clinically significant AS to SAVR and TAVR. Note: Plot of unadjusted time to AVR and associated means and difference in means (TAVR – SAVR).

Fig. 2 plots the unadjusted time, in days, from a patient's diagnosis of clinically signficant AS to AVR (within the included two-year window) and the associated means and difference in means (TAVR – SAVR). Out of the 14,225 patients with a record of AVR within two years of their clinically significant AS diagnosis, the average time to AVR was 157 days for TAVR and 98 days for SAVR.

### 3.3 Patient demographics and risk factors

Table 1 displays the patient demographics and risk factors for the overall sample of TAVR and SAVR cohorts. TAVR patients were older (mean age 76.19 versus 63.49), less male (57.63 % versus 70.59 %), and more likely to have Medicare insurance (69.88 % versus 34.94 %) as compared to SAVR patients. TAVR patients were also sicker than SAVR patients, with a higher average Elixhauser Comorbidity Score (6.44 versus 5.69) and a higher HFRS (9.57 versus 8.59).

**Table 1:** Patient demographics and risk factors.

		TAVR	SAVR	Total
Age (in years)		76.19 (8.34)	63.49 (11.46)	68.84 (12.03)
Male		3454 (57.63 %)	5811 (70.59 %)	9265 (65.13 %)
Caucasian race/ethnicity		4509 (92.85 %)	6446 (91.25 %)	10,955 (91.90 %)
Payor	Commercial	1670 (27.87 %)	4925 (59.83 %)	6595 (46.36 %)
	Medicare	4188 (69.88 %)	2876 (34.94 %)	7064 (49.66 %)
	Other	135 (2.25 %)	431 (5.24 %)	566 (3.98 %)
Region with the United States	Midwest	2818 (47.02 %)	4151 (50.43 %)	6969 (48.99 %)
	Northeast	1219 (20.34 %)	1491 (18.11 %)	2710 (19.05 %)
	South	1306 (21.79 %)	1974 (23.98 %)	3280 (23.06 %)
	West	650 (10.85 %)	616 (7.48 %)	1266 (8.90 %)
Elixhauser score		6.44 (2.71)	5.69 (2.84)	6.01 (2.81)
Hospital frailty risk score		9.57 (8.48)	8.59 (8.51)	9.00 (8.51)

### 3.4 Average number of cardiac imaging visits and cardiac specialist visits from clinically significant AS to AVR for TAVR and SAVR

Table 2 displays the estimated average number of cardiac specialist encounters and cardiac imaging events from clinically significant AS to AVR (SAVR or TAVR). On average, TAVR patients have almost twice as many cardiac specialist encounters (6.37 versus 3.73) and cardiac imaginig events (2.07 versus 1.18) than patients having a SAVR.

**Table 2:** Average number of cardiac specialist encounters and cardiac imaging events for patients receiving AVR (SAVR or TAVR).

	Number of AVRs	SAVR = 8232	TAVR = 5993
All cardiac specialist encounters (CS visits)			
	Mean (SD)	3.73 (5.37)	6.37 (7.28)

Number of cardiac imaging events (imaging)			
	Mean (SD)	1.18 (1.56)	2.07 (1.94)

Note: This may not include the imaging that led to the diagnosis of clinically significant AS. 100 % had at least one echo before their initial diagnosis, but some did not another echo before the AVR admission date. SD = standard deviation.

### 3.5 Average days to cardiac imaging visits and cardiac specialist visits from clinically signficant AS to AVR for TAVR and SAVR

**Fig. 3:**
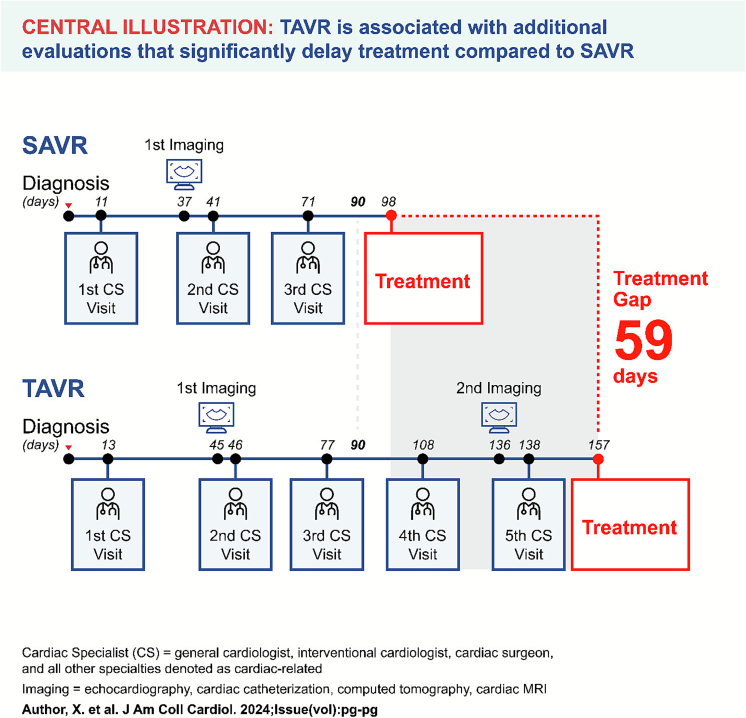
Average days to cardiac imaging visits and cardiac specialist visits from clinically significant AS to AVR for TAVR and SAVR. Note: Although average cardiac visits for SAVR were 3.73 and TAVR 6.37, the central figure is truncated to 3 visits and 5 visits for illustration purposes.

Fig. 3 provides a snapshot of a six-month timeline for TAVR and SAVR patients from time zero (their diagnosis of clinically signficant AS) to AVR through each cardiac specialist and cardiac specialist visit in chronocological order. The first visit from time zero is fairly consistent between TAVR and SAVR patients (11–13 days). However, beyond the first visit, the gaps between visits become larger for TAVR than for SAVR. This is especially the case for the second imaging visit, which was, on average, between four and five months from time zero for TAVR versus approximately 1.5 months from for SAVR. SAVR patients received their AVR after approximately three visits, while TAVR patients had ∼5 cardiac speciality visits before TAVR was performed. Within the six-month time horizion, TAVR patients on average had approximately twice as many cardiac speciality encounters than SAVR patients (3.73 [standard deviation or SD = 5.37] versus 6.37 [SD = 7.28] visits).

### 3.6 Model results for the difference in average days from diagnosis to AVR for patients receiving SAVR versus TAVR

Table 3 reports the adjusted regression models for differences in time to AVR as follows: (1) The first model only adjusts for demographics (age, sex, race, payor, region) and risk factors (Elixhauser Comorbidity Index and HFRS). (2) The second model keeps the demographics and risk factors in the model but then adds the number of cardiac specialist visits. (3) The third model also keeps the demographics and risk factors but adds the cardiac imaging visits. (4) Finally, the fourth model is a completely saturated model with demographics and risk factors + cardiac specialist visits + imaging. These modeling results clearly show how each factor impacts the differences in time to AVR between TAVR and SAVR. In the first model, when only adjusting for differences in baseline demographics and risk factors, TAVR patients waited on average 65 days longer than SAVR patients. In contrast, with use of the fully saturated model (4) which adds additional adjustment for the number of cardiac specialist and imaging visits, the TAVR/SAVR difference in time to AVR decreases from 65 to 11 days. This suggests that at least part of the longer delay to receiving TAVR is explained by pre-procedural encounters.

**Table 3:** Model results for the difference in average days from diagnosis to AVR for patients receiving SAVR versus TAVR.

Model	Adjustment variables	SAVR Mean	TAVR Mean	Difference (TAVR–SAVR)	Rate ratio (95 % CI)	P-value
1	Covariates demographics and risk factors only	84 days	149 days	65 days	1.77 (1.67–1.87)	<0.0001
2	Covariates demographics and risk factors + Cardiac specialist visits	89 days	115 days	26 days	1.29 (1.23–1.35)	<0.0001
3	Covariates demographics and risk factors + Imaging	92 days	113 days	21 days	1.24 (1.18–1.30)	<0.0001
4	Covariates demographics and risk factors + Cardiac specialist visits + Imaging	92 days	103 days	11 days	1.12 (1.07–1.17)	<0.0001

Longer TAVR delays explained, at least in part, by higher frequency of cardiac-related encounters. After adjusting for # cardiac specialist visits and imaging, the TAVR/SAVR difference in time to AVR decreases from 65 to 11 days. This suggests that at least part of the longer TAVR delay is explained by pre-procedural encounters.

### 3.7 Time to TAVR versus SAVR by subgroups of interest

Table 4 explores the different subgroups of interest (age, sex, race, payor, region, and cardiac specialist management/referral) to assess whether differences within subgroups explain delays in receiving TAVR. Regardless of subgroup, time to TAVR was always significantly longer than time to SAVR, as shown by all rate ratio 95 % confidence intervals greater than 1.0. For males, the magnitude of the difference between time to TAVR versus SAVR was greater than for females (64 days versus 47 days). For payor, the difference between TAVR and SAVR patients was greater for commerically insured patients than for Medicare patients (61 days versus 45 days). For those patients referred via a cardiac specialist, the magnitude was greater (60 days versus 55 days) than for those not managed/referred by a cardiac specialist.

**Table 4:** Time to TAVR versus SAVR by subgroups of interest.

	Sample size	Mean days to SAVR	Mean days to TAVR	Difference in mean days to AVR (TAVR-SAVR)	Rate ratio	Lower 95 % CI	Upper 95 % CI
		Unadjusted			Adjusted		
Age							
<65	5017	88	154	66	1.99	1.76	2.24
65–79	5905	110	163	53	1.82	1.69	1.96
80+	3303	103	152	49	1.73	1.52	1.97

Sex*							
Female	4960	108	155	47	1.63	1.50	1.78
**Male**	9265	**94**	**158**	64	1.85	1.73	1.98

Race							
Caucasian	13,548	99	157	58	1.75	1.65	1.86
Non-Caucasian	677	86	160	74	2.12	1.69	2.66

Region							
Midwest	6969	98	170	72	1.81	1.68	1.96
South	3280	112	156	44	1.73	1.56	1.92
Northeast	2710	90	136	46	1.66	1.48	1.85
West	1266	86	145	59	1.87	1.59	2.20

Payor*							
Medicare	7064	115	160	45	1.68	1.57	1.80
**Commercial**	6595	**89**	**150**	61	1.92	1.76	2.09
Other payer	566	88	149	61	1.73	1.31	2.28

Cardiac specialist management*							
**Yes**	8442	**85**	**145**	60	1.93	1.80	2.07
No	5783	118	173	55	1.58	1.46	1.71

Regardless of subgroup, Time to TAVR is always significantly longer than time to SAVR as shown by all rate ratio 95 % Confidence Intervals greater than 1.0. An asterisk (*) is displayed for Sex, Payer and Cardiac Specialist subgroups, which indicates a signficant interaction between the subgroup categories and AVR type (TAVR versus SAVR). For Sex, males are bolded because the magnitude of their difference between time to TAVR versus SAVR is greater than it is for females (64 days versus 47 days). For Payor, the magnitude of the difference in commerically insured patients is greater for TAVR versus SAVR than for Medicare patients (61 days versus 45 days). For those patients seeing a cardiac specialist, the magnitude is greater (60 days versus 55 days) than those who do not see a cardiac specialist.

## 4 Discussion

The present study, using real-world claims and EHR data, provides a comprehensive analysis of the time to AVR over a two-year time horizion in patients with clinically significant AS and compares the timelines between TAVR and SAVR. While previous research has established that longer wait times lead to worse outcomes, our findings uniquely identify the specific pre-procedural requirements that contribute to these delays. These results highlight systemic barriers in patients' journey of care and inequities in access to timely treatment depending on the treatment recommendation. Despite having the same disease that carries an equally grave prognosis, the type of treatment (TAVR versus SAVR) has a direct impact on patients' likelihood to derive benefit.

The primary finding of this study is the significant difference in the average time from diagnosis of clinically significant AS to AVR between TAVR and SAVR. Specifically, TAVR patients experienced a delay of approximately 59 days compared to SAVR patients (157 days versus 98 days, *p* < 0.0001). This delay is particularly concerning given the known risks associated with prolonged wait times in AS, including increased mortality and irreversible myocardial damage [2], which are exacerbated in patients with symptomatic severe AS [4,12].

Previous research indicates that even modest delays in AVR can significantly increase mortality rates, ranging from 2 % to 14 % [13]. A population-based study in Ontario, Canada, found that TAVR patients faced longer wait times than SAVR patients, which correlated with higher mortality and hospitalization rates [14]. Similarly, a US study during the COVID-19 shutdown reported that 10 % of TAVR patients experienced a cardiac event within the first month of waiting, with 35 % affected within three months [15]. Delays not only increase the risk of mortality and adverse events but are also associated with substantial healthcare costs. A cost-utility analysis from Spain showed that eliminating wait times for TAVR yields significant cost savings, particularly in patients with acute HF or syncope [16].

Our analysis underscores the impact of pre-procedural requirements, especially the higher frequency of cardiac specialist visits and pre-procedural imaging in TAVR patients, which partly explains the extended time to intervention. The consistent delay across all subgroups, regardless of age, sex, payor, or whether a cardiac specialist managed the referral, suggests systemic inefficiencies in the TAVR referral and evaluation process. Specifically, TAVR patients had, on average, 6.37 cardiac specialist encounters compared to 3.73 for SAVR patients, and 2.07 imaging visits compared to 1.18 for SAVR patients. These findings are consistent with prior studies indicating that the comprehensive screening required for TAVR, which often includes advanced imaging and multidisciplinary evaluations, can result in longer wait times compared to SAVR [12].

Our regression models further demonstrate that, after adjusting for demographics and risk factors, the difference in time to AVR between TAVR and SAVR decreases significantly when accounting for the number of cardiac specialist and imaging visits. In the fully adjusted model, the difference narrows to 11 days, suggesting that pre-procedural encounters significantly contribute to the longer time to AVR for TAVR patients. This finding is particularly important because it identifies specific, potentially modifiable factors that could be targeted to reduce delays in care delivery. Beyond the clinical consequences of delayed AVR, the increased pre-procedural requirements for TAVR impose substantial financial burdens on both patients and the healthcare system. Each additional specialist visit and imaging study incurs direct medical costs as well as indirect costs to patients including lost wages, transportation expenses, and caregiver time—burdens that are particularly significant for TAVR patients, who are typically older and averaged nearly twice as many encounters as SAVR patients.

Our findings extend existing literature that indicate these delays may stem from factors including referral patterns, disease recognition, patient preferences, systemic healthcare challenges, and the National Coverage requirements. Access to TAVR is particularly influenced by factors such as hospital ownership, size, and location. A study using data from the National Readmission Database (2012–2016) found that large, urban, not-for-profit teaching hospitals had higher TAVR utilization compared to smaller, nonurban, or investor-owned hospitals [17]. Despite the expansion of TAVR, rural patients and those in the Midwest or Southern US still face longer travel times to access care [18,19]. The National Coverage Determination for TAVR includes volume requirements on hospitals that can offer TAVR, coupled with intensive evaluation and procedural requirements. Based on historic data, these requirements are restrictive and contribute to inequitable access to TAVR. Such restrictive coverage constraints do not affect SAVR, so these significant barriers exclusively apply to one treatment modality for AS. A Transcatheter Valve Therapy registry study assessed TAVR outcomes during a period when these coverage restrictions were eased, and the results found that TAVR outcomes were maintained—suggesting that these requirements need to be reconsidered [20].

Subgroup analyses in our study indicate that the longer time to TAVR compared to SAVR is consistent across various patient demographics, including age, sex, race, region, and payer type. Notably, the difference in time to AVR is more pronounced in males and commercially insured patients. The delay asociated with commercially insured patients, as compared to Medicare patients, may be related to commerical payors' requirement to obtain pre-authorization for specialist visits, imaging tests, and the TAVR procedure itself. Additionally, patients referred by non-cardiac specialists experience longer delays on average for both TAVR and SAVR compared to those referred by a cardiac specialist; however, even those referred by a cardiac specialist experience longer delays for TAVR than SAVR, which highlights the potential impact of referral pathways on treatment timelines and the consistent delays in receving TAVR. These findings the multifaceted causes of delays in receiving AVR, encompassing both systemic healthcare inefficiencies and patient-specific factors.

These findings underscore the pressing need to scrutinize all components of the AS clinical pathway, including detection, diagnosis, referral, assessment/monitoring, and treatment recommendation. There is a critical need to recalibrate processes of care to reflect contemporary evidence, ensure seamless pathways, and guide patients in a timely way from the onset of symptoms to the best possible outcomes. Our results suggest that streamlining the pre-procedural requirements for TAVR, particularly the number of specialist visits and imaging events, could substantially reduce wait times and potentially improve patient outcomes. We are not proposing fewer diagnostic modalities—the clinical information required for TAVR remains unchanged. Rather, the target is reducing distinct encounters through approaches such as ordering CT at the time of referral, coordinating imaging within single-visit evaluations, and using coronary CT in lieu of invasive angiography when appropriate. This is particularly important from the patients' perspective, as repeat consultations and other clinical requirements are associated with significant burdens for patients to secure the financial, social, and physical resources required [21,22].

In the first decade of TAVR availability, coverage requirements for two separate evaluations—one by an interventional cardiologist and the other a cardiac surgeon—helped ensure appropriate TAVR treatment selection. However, given the widespread adoption of the multidisciplinary team approach across cardiac programs, improvements in the device and procedure, and evolving indications across all patient surgical risk levels, it is possible that the coverage requirement for repeat consultations conttributes to delays in access to care and increasing burden on the healthcare system. For the majority of contemporary transfemoral TAVR candidates, a single in-person clinical evaluation by either specialist can establish patient suitability, with subsequent heart-team assessment and shared decision-making occurring through telemedicine consultations, concurrent multidisciplinary review, or formal case conferences. This approach preserves the integrity of the heart-team model while reducing scheduling delays that may negatively impact patient outcomes [21,22], and may be negatively impacting patient outcomes as a result of the additional time required to schedule and conduct these clinical assessments. In contrast, for patients who are not a candidate for transfemoral TAVR, alternate approach TAVR procedures may warrant evaluation by a cardiac surgeon due to their specialized knowledge of anatomical suitability for these more complex procedures. Given the critical nature of timely intervention in severe AS, more efficient integration of multidisciplinary evaluations and advanced imaging may help optimize the pre-procedural process. This suggestion is supported by evidence that timely AVR, whether via SAVR or TAVR, markedly enhances survival rates, alleviates symptoms, and improves quality of life [4,12]; and that delay in AVR is associated with worse outcomes and increased costs post-AVR [2].

Leveraging best practices for TAVR programs to streamline the patient journey can also reduce the time to treatment. This involves implementing efficient clinical pathways and adopting a minimalist approach to both peri-procedural and pre-procedural stages, which can reduce resource demands and expand program capacity. Additional innovative strategies are essential to eliminate barriers and expedite treatment, including:

• **Patient-Centered Assessments**: Prioritizing patient needs through approaches such as single-visit evaluations, patient navigator, and satellite clinics to minimize travel time and accommodate individual preferences.
• **Expanded Virtual Care**: Increasing the use of telemedicine and remote monitoring to enhance accessibility and convenience for patients.
• **Advanced Data Analytics**: Applying data to map clinical workflows, automate routine tasks, identify bottlenecks, and support decision-making with evidence-based insights.

Collectively these strategies, TAVR programs can augment their capacity, improve patient access, and alleviate the overall burden on patients throughout their care journey. While our findings provide important insights into the mechanisms of delay in AVR delivery, several limitations of our study should be considered when interpreting these results.

### 4.1 Limitations

This study has several limitations. The retrospective design and reliance on claims and EHR data may introduce biases related to data accuracy and completeness. Additionally, the study does not account for all potential confounders, such as patient preferences and provider-level factors, which may influence the timing of AVR. Furthermore, site-level confounders such as hospital type, size, and whether the site was a large referral center versus a community program were not accounted for in this model, nor were data on waitlist mortality available. This analysis excludes patients with AS who did not undergo AVR within two years. It is likely that the same factors causing delays in treatment for the patients included in this study also prevented others from accessing treatment altogether; however this important group is not accounted for the current analysis.Finally, we limited our analysis to patients with documented clinically significant AS diagnosis dates (60 % of all AVR patients in the database), as this was necessary to calculate our primary outcome of time to treatment. Patients without clear documentation of their symptomatic AS diagnosis date were excluded, which may limit generalizability.

## 5 Conclusions

This study highlights the significant delay in time to AVR for patients in the United States undergoing TAVR as compared to SAVR, and shows that the delay is primarily driven by more extensive pre-procedural requirements. Our findings demonstrate that the higher frequency of cardiac specialist visits and imaging events account for approximately 83 % of the observed delay difference between TAVR and SAVR (reducing the gap from 65 days to 11 days when adjusted for these factors). These results move beyond simply documenting that delays exist to identifying specific, potentially modifiable factors in the care pathway that could be targeted for improvement. Future research should focus on identifying specific strategies to reduce pre-procedural delays and exploring the impact of these interventions on patient outcomes.

## CRediT authorship contribution statement

**Curtiss Stinis:** Writing – review & editing, Writing – original draft, Validation, Supervision, Methodology, Investigation, Formal analysis. **Sean Tunis:** Writing – review & editing, Validation, Methodology, Investigation, Conceptualization. **Sandra Lauck:** Writing – review & editing, Validation, Supervision, Formal analysis, Conceptualization. **Aakriti Gupta:** Writing – review & editing, Validation, Supervision, Investigation, Conceptualization. **Shannon Murphy:** Writing – review & editing, Writing – original draft, Visualization, Validation, Software, Resources, Methodology, Investigation, Formal analysis, Data curation, Conceptualization. **Soumya Chikermane:** Writing – review & editing, Writing – original draft, Validation, Supervision, Project administration, Methodology, Investigation, Funding acquisition, Formal analysis, Data curation, Conceptualization. **Seth Clancy:** Writing – review & editing, Supervision, Resources, Investigation, Funding acquisition, Data curation, Conceptualization. **Mark Russo:** Writing – review & editing, Writing – original draft, Validation, Supervision, Methodology, Investigation, Conceptualization.

## Funding

This study was funded by 10.13039/100006520Edwards Lifesciences.

## Declaration of competing interest

The authors declare the following financial interests/personal relationships which may be considered as potential competing interests: Dr. Curtiss Stinis is a consultant for and receives honorarium from Edwards Lifesciences, Medtronic, Shockwave Medical, and Boston Scientific; Dr. Sean Tunis is a Senior Fellow at the Center for the Evaluation of Value and Risk in Health at the Institute for Clinical Research and Health Policy Studies at Tufts Medical Center. The center receives funding from government, private foundations, and pharmaceutical industry sources. He also consults with multiple pharmaceutical, medical device, and diagnostic companies including Edwards Lifesciences; Dr. Sandra Lauck is a consultant for Edwards Lifesciences and Abbott. Dr. Aakriti Gupta is a co-founder of Heartbeat Health, Inc. (a telehealth cardiology company) and icardio.ai (an artificial intelligence echocardiography company); Shannon Murphy, Dr. Soumya Chikermane and Seth Clancy are all employees of Edwards Lifesciences; Dr. Mark Russo received research grants from Edwards Lifesciences, Jena Valve, Abbott, and has served as a consultant or advisor for Abbott, and Edwards Lifesciences.
